# Characterization and comparison of the digestive physiology of two scombrids, *Katsuwonus pelamis* and *Sarda sarda*, in the Gulf of Cádiz

**DOI:** 10.1371/journal.pone.0249541

**Published:** 2021-04-14

**Authors:** Diogo Dias, Gian Marco Dardengo, Sofia Engrola, Carmen Navarro-Guillén

**Affiliations:** Aquaculture Research Group, Centro de Ciências do Mar do Algarve (CCMAR), Faro, Portugal; Universidad de Cádiz, Facultad de Ciencias del Mar y Ambientales, SPAIN

## Abstract

Fish and other marine animals have a unique and intimate interaction with their surrounding environment. Diet type or trophic level play significant roles in modulating species digestive physiology. However, little is known about how the trophic niche influences digestive activity and gut microbiota in scombrids species. The aim of the present study was to analyse and describe the digestive physiology of the skipjack tuna (*Katsuwonus pelamis*) and the Atlantic bonito (*Sarda sarda*) as bioindicator of the feeding ecology and trophic niche for both species in the Gulf of Cádiz (Spain). For that, fish proximate composition, pH of digestive organs and digestive enzymes activity levels were analysed in 10 individuals of each species to gain insight into the digestive physiological adaptations of the two species of scombrids. In addition, intestinal microbiota composition was determined for the skipjack tuna. The integration of the results suggested a clear trophic niche segregation between both species. Stomach pH was associated in both species with infrequent feeding events. Body proximate composition and digestive lipases activity patterns pointed to a high predominance of lipids in the Atlantic bonito diet, suggesting oily fish as main prey. On the other hand, results supported the skipjack tuna as a highly opportunistic feeder with a more varied diet, including fish but also crustaceans as preys. The gut microbial community in the latter species is dominated by Firmicutes and Tenericutes at phylum level, and by Mycoplasma, Blautia and Dorea at genus level. The present study contributes to advance the knowledge on the feeding behaviour and physiology diversity in fish species as a result of adaptation to a particular habitat.

## Introduction

Fish, the group with the largest taxonomic and ecological diversity in vertebrates, have a unique and intimate interaction with their surrounding environment [1]. Environmental conditions, mainly temperature and diet type or trophic level, play significant roles in modulating species digestive physiology. The available biotope, preys and the predators are delimited by the geographic location, ecology, and climate. To face the wide spectrum of conditions in the oceans, depending on their spatial variability, species have evolutionary adapted their feeding behaviour and physiology to the specific conditions of each particular habitat [2]. Therefore, individual’s ability to digest and assimilate the nutrients to thrive will depend on both, the ingested feed, and the capacity to modulate and adapt its digestive enzymes and metabolic processes [3,4].

The high versatility in fish feeding habits is usually reflected in different anatomical and functional features. This allows fish to explore a wide range of feed resources and maximize its availability in the environment [5]. The rapid adaptation to the variations related to prey quality and availability is possible due to a highly dinamic digestive system functionality. The main role of the digestive system is the digestion and assimilation of nutrients. The presence of nutrients in the gastrointestinal tract (GIT) is the main stimulus for digestive enzymes secretion, and at the same time, enzymes quantity and activity are modulated by the feed composition and digestibility [6]. In this respect, fish have a high plasticity in digestive enzymes production in response to diet, since the metabolic cost associated to the enzymatic production might be wasted in those individuals consuming low levels of the corresponding substrates. For this reason, the analysis of digestive enzymes activity in fish can be used as nutritional biomarker and as indicator of their feeding ecology and trophic niche for a specific habitat [7].

In the last years, several studies have focused on the intestinal microbiota due to findings that microbes residing in the intestine have a key role in the host global metabolism. In vertebrates, the microbial structure and composition, as well as its ecological function, are strongly influenced by a wide variety of factors, including host genetics, natural habitat, diet and phylogeny [8]. However, despite the increasing attention on this topic, there is scarce information addressing how the intestinal bacterial community is modulated by diet and temperature changes linked to habitat transitions. To our knowledge, the only studies assessing how habitat transition modulates intestinal microbial composition has been performed in the Atlantic salmon (*Salmo salar*) [9,10]. The migration from freshwater to seawater affects alpha- and beta-diversity, as well as the number of operational taxonomic units (OTUs), being all these parameters higher in freshwater fish. However, both studies identified a core microbiota in all fish, indicating that certain microbes are kept to ensure minimal functions within the GIT.

The skipjack tuna (*Katsuwonus pelamis*) and the Atlantic bonito (*Sarda sarda*) are two species belonging to the Scombridae family with notable commercial importance. Despite this, few studies have focused on the trophic biology of the species. The skipjack tuna is a highly migratory species distributed in warm and temperate offshore waters, being an opportunistic predator with diet variations both spatially and temporally [11]. Specifically, for Spanish marine areas (Balearic Sea, Alboran Sea and Gulf of Cadiz), diet composition is significantly varied among regions; from North Atlantic krill (*Meganyctiphanes norvegica*) as main prey in the Balearic Sea to anchovy (*Engraulis encrasicolus*) in the Gulf of Cádiz [12]. On the other hand, the Atlantic bonito occurs in pelagic coastal waters on both sides of the Atlantic Ocean, Gulf of Mexico, Mediterranean and Black Sea. In Atlantic and Mediterranean coasts near the Strait of Gibraltar, the Atlantic bonito feeds preferably on schooling fish such as sardines, anchovies and mackerel, and other pelagic teleost fishes [13,14]. The information available on the digestive physiology of both species is scarce, being of great interest to improve the knowledge about the nutritional metabolism of these fast-growing species with spatial variability.

The aim of the present study was to analyze and describe the digestive physiology of the skipjack tuna and the Atlantic bonito in the Gulf of Cádiz (Spain). For this purpose, fish proximate composition and digestive enzyme activities were analyzed in both species to gain insight into the digestive physiological adaptations of the two species of scombrids to a specific habitat. In addition, intestinal microbiota composition was determined for the skipjack tuna.

## Materials and methods

### Sampling

A total number of 10 fish for each species were sampled in August 2019 in the Gulf of Cádiz. All specimens were caught by recreational trolling boats during daylight hours. The fish were measured to the nearest cm (straight fork length, SFL) and weighed to the nearest g (total weight, W) (Table 1). Digestive systems and a muscle sample before the caudal peduncle were collected from all fish. Samples were stored at -20 °C until further analysis.

**Table 1:** Fish biometric data.

	SFL (cm)	W (g)
*Katsuwonus pelamis*	37.5 ± 3.7	939.3 ± 251.7
*Sarda sarda*	43.6 ± 1.9	1093.9 ± 162.3

SFL, straight fork length; W, total weight.

Summary of biometric data for skipjack tuna (*Katsuwonus pelamis*) and the Atlantic bonito (*Sarda sarda*) (mean ± SD).

At the laboratory, fish digestive systems were thawed and, in the case of *K*. *pelamis*, the distal intestine of five samples were aseptically dissected and immediately transferred to DNA/RNA Shield^™^ tubes (Zymo Research, Irvine, CA) and sent for microbiota composition analysis. The remaining samples were processed for enzymatic analysis as described below.

### Body proximate composition

At the laboratory the tissue samples were thawed, severed into small pieces with an electric knife, ground in a mincer until sample was homogeneous and frozen at -80°C for at least 12 h. A sub-sample was taken for protein and lipid determination, freeze-dried and ground with an electric grinder until an apparently homogeneous powder was obtained. The samples were sieved using a 1000μm sieve to remove the unmilled particles, ground with a pestle and mortar until they were completely homogeneous.

For moisture determination, the crucibles with the individual samples were dried at 100°C for 24h in an oven and weighed. Afterwards, the same crucible was combusted in a muffle furnace for 12 h at 550°C, and the obtained ashes were weighed. Fish moisture and ash were determined in triplicate for each individual. Moisture and ash contents were calculated as follow:

$$Moisture\;(\%)=100\;-\;[(\frac{sample\;dried\;weight\;(g)}{sample\;wet\;weight\;(g)})\;x\;100]$$

$$Ashes\;(\%)=(\frac{sample\;ashes\;weight\;(g)}{sample\;wet\;weight\;(g)})\;x\;100$$

Protein was determined following the method of Lowry modified by Rutter [15], based on two reactions: the Biuret reaction, where peptide bonds of proteins, under alkaline conditions, react with cooper to produce Cu+ that reacts with the Folin reagent; the Foloin-Ciocalteau reaction where phosphomolybdotungstate is reduced to heteropoly-molybdenum blue by copper-catalyzed oxidation of aromatic amino acids.

Lipid analysis was determined using a modified method by Bligh and Dyer [16]. In short, this method consists in the homogenization of tissue with a mix of chloroform and methanol. Followed by a dilution and water that separates the homogenate in 3 layers, with the chloroform layer containing the lipid contents. The purified lipid extract was obtained by the isolation of the chloroform layer. This layer was then evaporated to dryness and the weight of lipid residue was determined.

### Digestive enzymes activity measurement

Prior to enzymatic analysis, digestive system samples were divided into stomach (ST), caecal mass (CM) and intestine (INT). Firstly, a visual evaluation for presence of feed within the stomach and intestine was performed to establish the feeding status of the fish. After, digestive organs were weighed to the nearest 0.1 g.

The weight relevance of each organ was verified with the organ-somatic index (OSI), calculated according to the following formula:

$$OSI\;(\%)=\frac{weight\;of\;the\;organ\;(g)}{weight\;of\;the\;fish\;(g)}\;x\;100$$

For digestive enzyme activities measurement, intestine samples were dissected in proximal, middle, and distal intestine (PI, MI and DI, respectively) (S1 Fig). Each section of the digestive system was then homogenized in 30, 20 or 5 ml (for stomach, caecall mass and intestine segments, respectively) of distilled water with the use of an Ultra-Turrax^®^ Homogenizer T-18 (IKA-Werke) and pH was measured for all samples (pHSpear, Thermo Scientific Eutech Instruments). Samples were centrifugated at 4°C, 3500 g, 30 min, and the supernatant was collected and stored at -20 °C until further analysis. All samples were kept in ice during the process described above to avoid enzymes denaturation and/or damage. Concentration of soluble protein in extracts was determined by Bradford method [17].

For the characterization and comparison of the digestive function in both species the specific activity of the following enzymes was analysed: pepsin and acidic chitobiase in the stomach and, total alkaline proteases, amylase, lipase and alkaline chitobiase in the caecal mass and intestine sections (IP, IM and ID). Since enzymes are highly sensitive to changes in pH, the buffers used for analysis were adjusted to the pH values measured in each of the digestive organs, thus ensuring the results were close to the enzymatic activity at sampling point. Table 2 shows the buffers’ pH values used for enzymatic analysis for each species and digestive organ.

**Table 2:** pH values used for enzymatic analysis.

	Stomach	Caecal mass	Intestine
*K*. *pelamis*	6.75	6.75	7.35
*S*. *sarda*	6.60	6.60	6.95

pH values of buffers used for the measurement of enzymatic activity for each species and digestive section.

Since gastric digestive enzymes are dependent on acidic conditions to function, the enzymes tested in the stomach were tested at the pH measured in the samples but also at pH 3 to force the activation of precursors (namely pepsinogen), thus determining both, active and total (pepsin and pepsinogen) enzymatic activity.

Pepsin activity was determined by the method of Anson [18]: 15 μl of extract was mixed with 1 ml of 0.5% acid-denatured bovine hemoglobin (Sigma Aldrich) diluted in 0.2 M HCl-glycine or citrate-phosphate buffers for total or active pepsin, respectively. After incubation at room temperature for 20 min, the reaction was stopped by adding 0.5 ml of 20% trichloroacetic acid (TCA, VWR), cooled to 4 °C for 15 min and then centrifuged at 11000 g for 15 min at 4°C. The absorbance of the resulting supernatant was measured at 280 nm. Blanks were constructed by adding the enzyme extracts to the reaction mixture just after the TCA addition.

Chitobiase activity (both acidic and alkaline) was assayed through a modification of German et al. [19] protocol. The fluorogenic substrate 4-methylumbelliferyl-N-acetyl-d-glucosaminide (69585, Sigma-Aldrich) was dissolved in 0.2 M HCl-glycine buffer, citrate-phosphate or 50 mM Tris-HCl buffer for acidic (total and active) or alkaline activity, respectively, to a final concentration of 200 μM. For analysis, 90 μl of substrate and 15 μl of tissue homogenate were added to the microplate. Fluorescence was measured at 365 nm (excitation) and 450 nm (emission).

Total alkaline proteases activity was measured by modification of Anson [18]; mixing 15 μl of extract with 1 ml of 1% casein (Sigma Aldrich) diluted in citrate-phosphate buffer. After incubation at room temperature for 20 min, the reaction was stopped by adding 500 μl of 20% trichloroacetic acid (TCA, VWR), cooled to 4 °C for 15 min and then centrifuged at 11000 g for 15 min at 4°C. The absorbance of the resulting supernatant was measured at 280 nm. Blanks were constructed by adding the enzyme extracts to the reaction mixture just after the addition of TCA.

Amylase activity was determined by the 3,5-dinitrosalicylic acid (DNS) procedure [20], using 2% soluble starch diluted in Tris-HCl buffer as substrate. 30 μl of tissue extract and 300 μl of substrate were incubated at 37 °C for 30 min. The reaction was stopped by the addition of 150 μl DNS and was heated in boiling water for 10 min. Then, after cooling in ice, 1.5 ml of distilled water was added to the mixture and the absorbance was measured at 540 nm. Blanks were constructed by adding the enzyme extracts to the reaction mixture just after the addition of DNS.

Lipase activity was measured using the reactive substrate 4-methylumbelliferyl oleate (SIGMA M75164), diluted to a final concentration of 0.4 mM in phosphate buffer. For the analysis 15 μl of sample homogenate were loaded into a microplate followed by 250 μl of substrate, following the protocol of Rotllant et al. [21]. Fluorescence was measured at 355 nm (excitation) and 460 nm (emission).

Pepsin, alkaline proteases and amylase activities were indicated in activity unites (U) per mg of protein. For proteases, one activity unit was defined as the amount of enzyme that was required to hydrolyze hemoglobin or casein to give 1 μg of tyrosine in 1 min. For that, the molar extinction coefficient of tyrosine in each buffer and pH used was calculated. For amylase, one activity unit was defined as the amount of enzyme that is required to hydrolyze starch to give 1 μg of maltose in 1 min. For that, the molar extinction coefficient of maltose in Tris-HCl buffer at each pH used was calculated. For the remaining enzymes analyzed, for which fluorogenic substrates were used, the liberation of the fluorophore was kinetically followed, and activities were expressed as relative fluorescence units (RFU) per mg of protein.

### *Katsuwonus pelamis* gut microbiota composition

The samples were processed and analyzed with the ZymoBIOMICS^®^ Targeted Sequencing Service for Microbiome Analysis (Zymo Research, Irvine, CA).

The ZymoBIOMICS^®^-96 MagBead DNA Kit (Zymo Research, Irvine, CA) was used to extract DNA using an automated platform. Bacterial 16S ribosomal RNA gene targeted sequencing was performed using the Quick-16S^™^ NGS Library Prep Kit (Zymo Research, Irvine, CA). The bacterial 16S primers amplified the V3-V4 region of the 16S rRNA gene. These primers have been custom-designed by Zymo Research to provide the best coverage of the 16S gene while maintaining high sensitivity. The sequencing library was prepared using an innovative library preparation process in which PCR reactions were performed in real-time PCR machines to control cycles and therefore limit PCR chimera formation. The final PCR products were quantified with qPCR fluorescence readings and pooled together based on equal molarity. The final pooled library was cleaned with the Select-a-Size DNA Clean & Concentrator^™^ (Zymo Research, Irvine, CA), then quantified with TapeStation^®^ (Agilent Technologies, Santa Clara, CA) and Qubit^®^ (Thermo Fisher Scientific, Waltham, WA). The final library was sequenced on Illumina^®^ MiSeq^™^ with a v3 reagent kit (600 cycles). The sequencing was performed with 10% PhiX spike-in.

The ZymoBIOMICS^®^ Microbial Community Standard (Zymo Research, Irvine, CA) was used as a positive control for each DNA extraction. The ZymoBIOMICS^®^ Microbial Community DNA Standard (Zymo Research, Irvine, CA) was used as a positive control for each targeted library preparation. Negative controls (i.e. blank extraction control, blank library preparation control) were included to assess the level of bioburden carried by the wet-lab process.

### Data analysis

Percentage data (proximate composition and OSI) were arcsine square root-transformed prior to analysis. Differences in body proximate composition and OSI between species were assessed by means of unpaired two-tailed Student’s t–test. Data from organs’ pH values and enzymatic activity levels were subjected to a two-way ANOVA, considering fish species and digestive organs as variables. Following the two-way ANOVA and, whenever significant differences were identified, means were compared by the Post hoc multiple comparisons Tukey’s test or by means of unpaired two-tailed Student’s t–test, for differences between digestive organs or fish species, respectively. Before analyses, the ANOVA assumptions of homogeneity of variances was tested using the Levene’s test. Analyses were performed with SPSS 26 software (IBM, New York, USA). The differences among treatments were considered significant at P < 0.05. All descriptive statistics are expressed as mean ± standard deviation of the mean (SD). The similarities between body proximate composition of the samples from both species were represented in a Principal Component Analysis using Primer V7 software (PRIMER-E Ltd., Auckland, NZ).

For bioinformatics analysis of gut microbiota composition, unique amplicon sequences variants were inferred from raw reads using the DADA2 pipeline [22]. Chimeric sequences were also removed with the DADA2 pipeline. Taxonomy assignment was performed using Uclust from Qiime v.1.9.1 with the Zymo Research Database, a 16S database that is internally designed and curated, as reference. Composition visualization, alpha-diversity, and beta-diversity analyses were performed with Qiime v.1.9.1 [23]. If applicable, taxonomy that have significant abundance among different groups were identified by LEfSe [24] using default settings.

## Results

### Biometrics measurements

**Fig 1:**
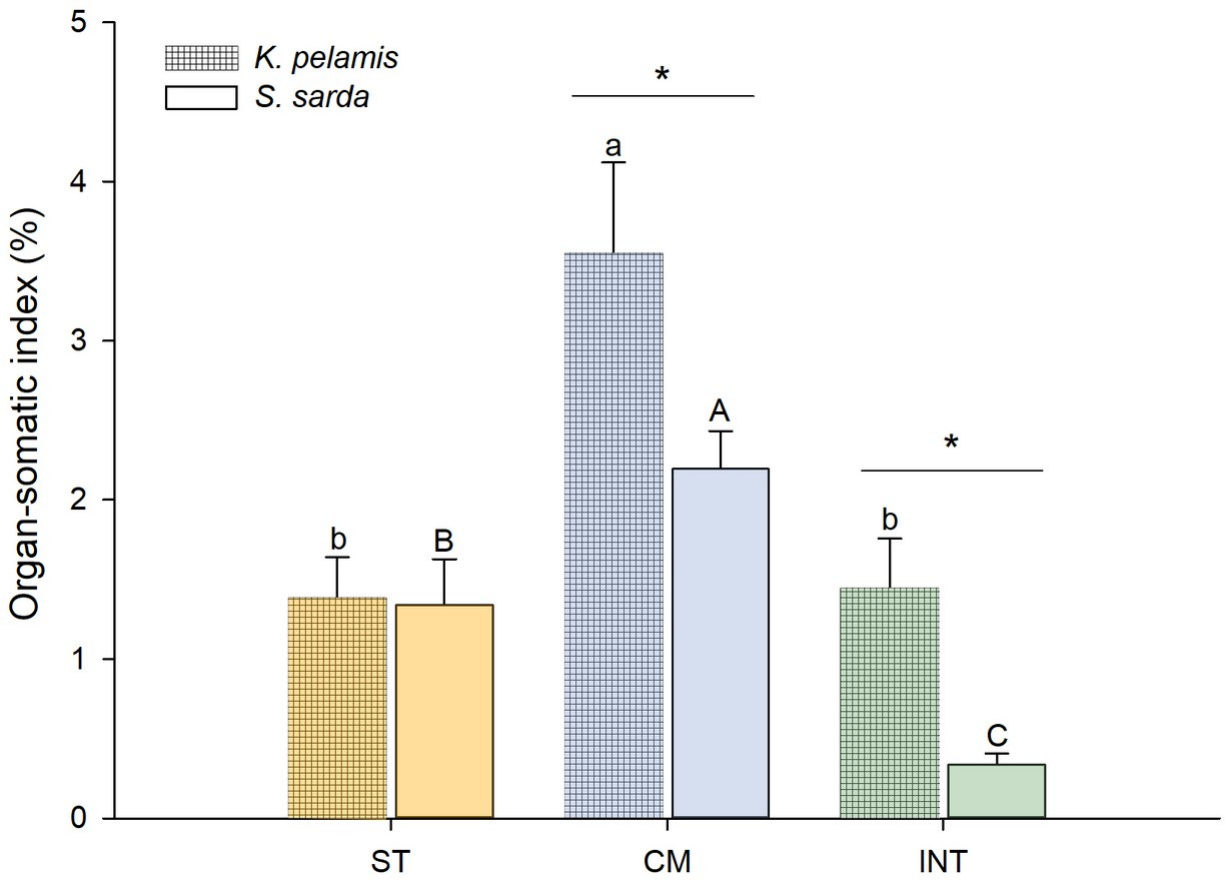
Digestive organ-somatic indices. Organ-somatic indices (OSI) for stomach (ST, orange), caecal mass (CM, blue) and intestine (INT, green) of *Katsuwonus pelamis* (gridded bars) and *Sarda sarda* (smooth bars). Data are presented as mean ± SD (n = 10). Different lowercase letters (a,b) represent statistical differences between digestive organs of *S*. *sarda*, while different capital letters (A, B, C) represent statistical differences between digestive organs of *K*. *pelamis* (P<0.05). Asterisks represent statistical differences between species (P<0.05). Absence of asterisk indicates no statistical difference between species (P>0.05).

All fish showed similar patterns of gastrointestinal content, with empty stomachs and full intestines, suggesting similar feeding status (S1 Table). The organ-somatic indices (OSI) results (Fig 1) revealed that the digestive organ with the highest mass in proportion to total body mass was the CM for both species (2.19 ± 0.29% and 3.54 ± 0.26% for *S*. *sarda* and *K*. *pelamis*, respectively) (P = 0.000). Specifically, for *K*. *pelamis*, stomach and intestine did not show differences in their organ-somatic indices (1.38 ± 0.26% and 1.44 ± 0.31%, respectively). On the other hand, for *S*. *sarda*, stomach was significantly higher than the intestine as proportion of the total body mass (1.34 ± 0.29% and 0.34 ± 0.07%, stomach and intestine, respectively). When comparing the two species, *K*. *pelamis* showed statistically higher somatic indices for the caecal mass and intestine than *S*. *sarda* (P = 0.000 and 0.000, respectively).

**Fig 2:**
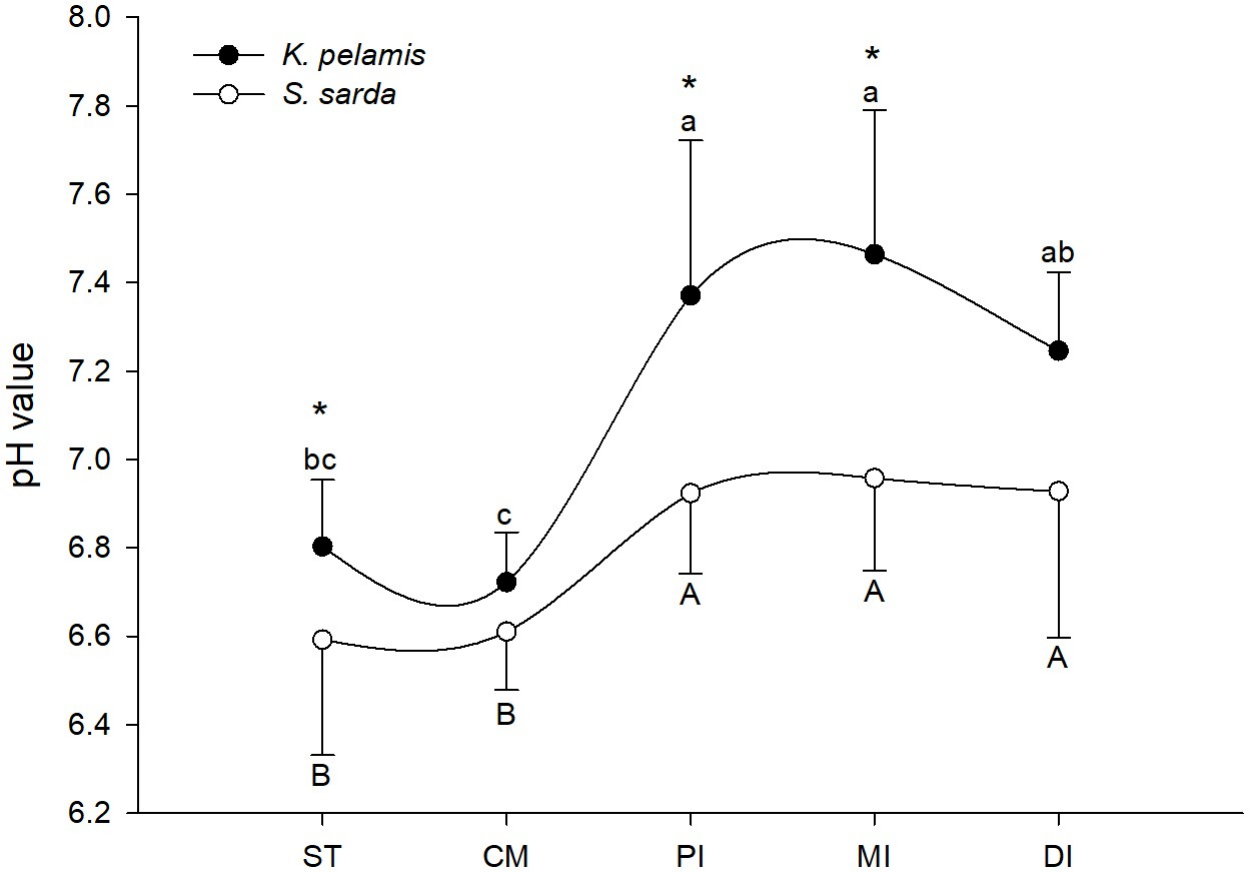
Digestive organs pH values. pH values of stomach (ST), caecal mass (CM), proximal intestine (PI), middle intestine (MI) and distal intestine (DI) of *Katsuwonus pelamis* (black dots) and *Sarda sarda* (white dots). Data are presented as mean ± SD (n = 10). Different lowercase letters (a, b, c) represent statistical differences between digestive organs of *S*. *sarda*, while different capital letters (A, B) represent statistical differences between digestive organs of *K*. *pelamis* (P<0.05). Asterisks represent statistical differences between species (P< 0.05). Absence of asterisk indicates no statistical difference between species (P>0.05).

pH values measured along the digestive tract were always close to the neutral value for the two species, with mean values of 7.12 ± 0.22 and 6.8 ± 0.22 for *K*. *pelamis* and *S*. *sarda*, respectively (Fig 2). pH levels of digestive organs showed a general trend to higher values on *K*. *pelamis* compared to *S*. *sarda*, becoming statistically different for stomach and proximal and middle intestine (P = 0.040, 0.002 and 0.001, respectively). For *K*. *pelamis* the highest pH values were recorded in the proximal and middle intestine, while the lowest pH values were measured in the caecal mass and stomach (P = 0.000), while *S*. *sarda* showed statistical higher pH values in all intestine segments when compared to stomach and caecal mass (P = 0.000).

### Body proximate composition

Body proximate composition of *K*. *pelamis* and *S*. *sarda* is shown in Table 3. No significant differences between species were found for the moisture, ash and protein content (P = 0.216, 0.245 and 0.877, respectively). By contrast, the lipids content was statistically higher in *S*. *sarda* (P = 0.000).

**Table 3:** Body proximate composition.

Proximate composition (% BW)	*K*. *pelamis*	*S*. *sarda*
Moisture	70.00 ± 1.14	69.15 ± 1.09
Protein	23.19 ± 2.47	22.98 ± 1.44
Lipids	1.61 ± 0.63^b^	3.51 ± 0.53^a^
Ash	5.19 ± 1.13	4.53 ± 0.55

Data are presented as mean ± SD (*n* = 6). Different letters (a,b) represent statistical differences between species (P< 0.05). Absence of letters indicates no statistical difference between species (P>0.05).

Body proximate composition of *Katsuwonus pelamis* and *Sarda sarda* (% BW).

**Fig 3:**
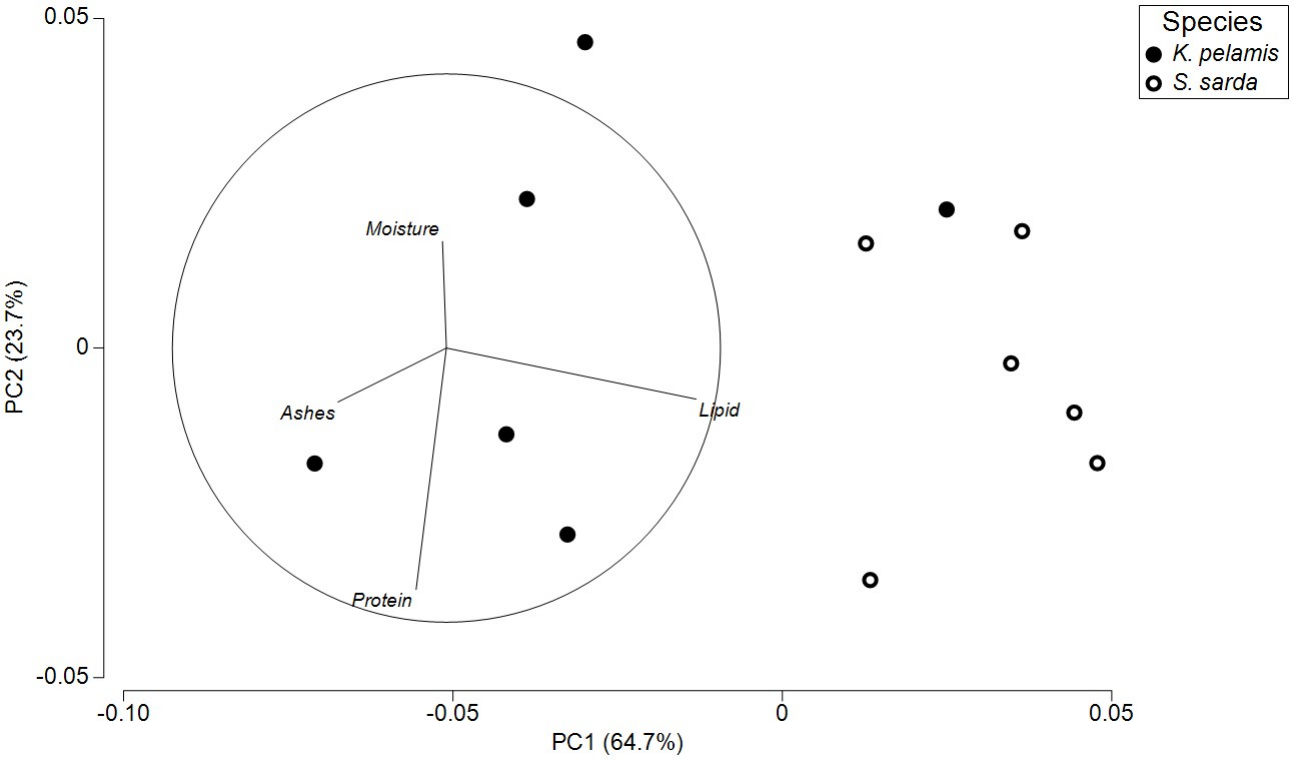
Body composition PCA analysis. Principal Component Analysis (PCA) of body proximate composition of *Katsuwonus pelamis* and *Sarda sarda*.

Principal component analysis (PCA) plot demonstrated a clear separation between species in body proximate composition, with a single *K*. *pelamis* sample appearing separate from the main cluster (Fig 3). Component 1 explained 64.7% of the analysis, while component 2 explained 23.7%. Vectors’ circle confirmed lipids content as the main factor clustering the samples.

### Digestive enzyme activities

**Fig 4:**
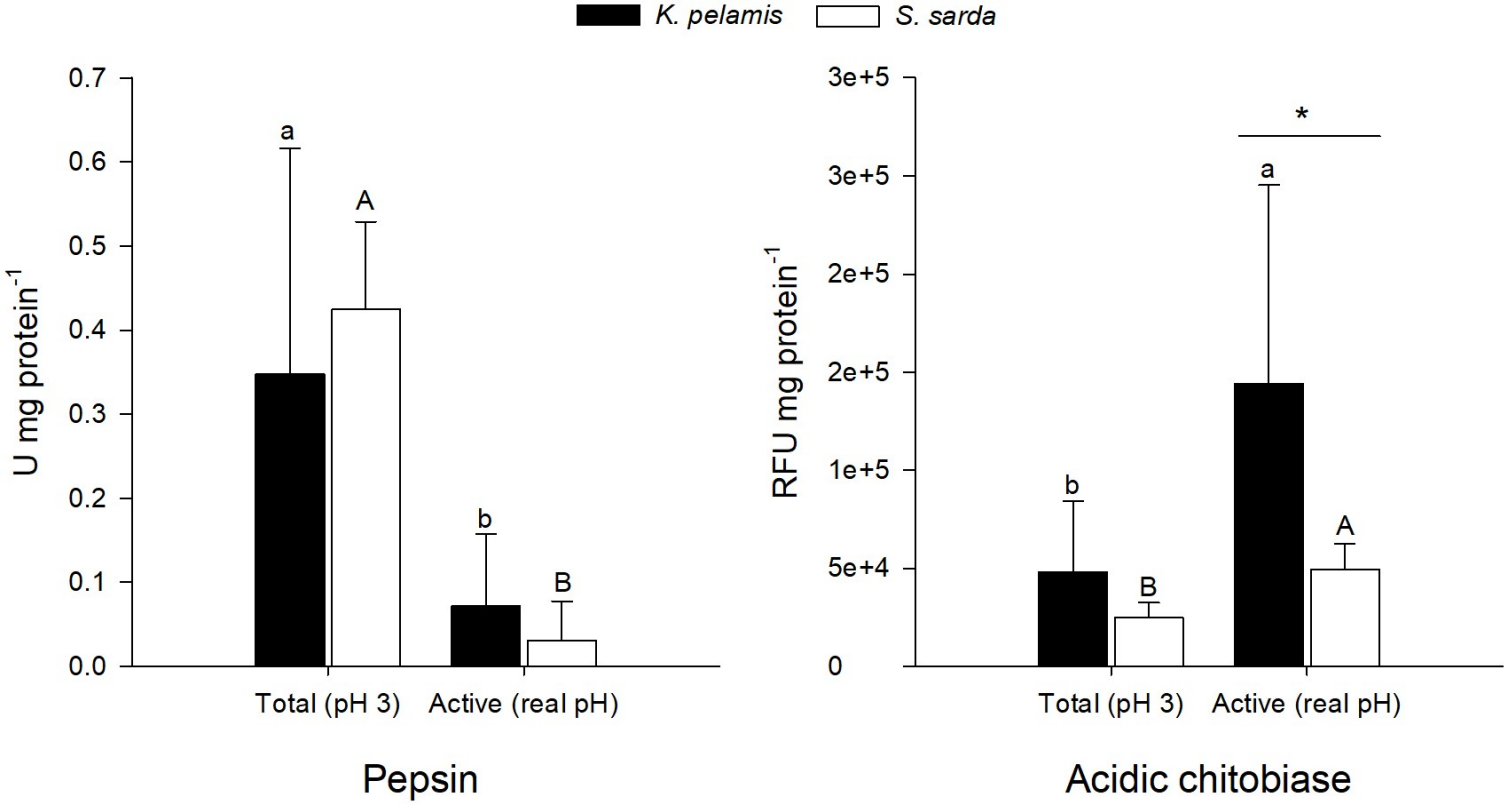
Acidic digestive enzymes activity levels. Total and active enzymatic levels of pepsin and acidic chitobiase in *Katsuwonus pelamis* (black dots) and *Sarda sarda* (white dots) stomachs. Data are presented as mean ± SD (*n* = 10). Different lowercase letters (a,b) represent statistical differences between digestive organs of *S*. *sarda*, while different capital letters (A, B) represent statistical differences between digestive organs of *K*. *pelamis* (P<0.05). Asterisks represent statistical differences between species (P< 0.05). Absence of asterisk indicates no statistical difference between species (P>0.05).

The results of the acidic enzymes, pepsin and acidic chitobiase, are shown in Fig 4. As expected, total pepsin (pH 3) was statistically higher than active pepsin at sampling point (analysis performed at stomach pH value) for both species (P = 0.030 and 0.000, *K*. *pelamis* and *S*. *sarda*, respectively). Pepsin activity levels were similar between both species at both, acidic and sampling conditions (P = 0.467 and 0.318, respectively). By contrast, acidic chitobiase activity was statistically higher at measured pH value in the stomach than at acidic conditions both for species (P = 0.034 and 0.000 for *K*. *pelamis* and *S*. *sarda*, respectively). Acidic chitobiase levels were similar between both species in acidic conditions (P = 0.111), while at higher pH values (real pH) *Katsuwomus pelamis* showed higher activity levels than *Sarda sarda* (P = 0.034).

**Fig 5:**
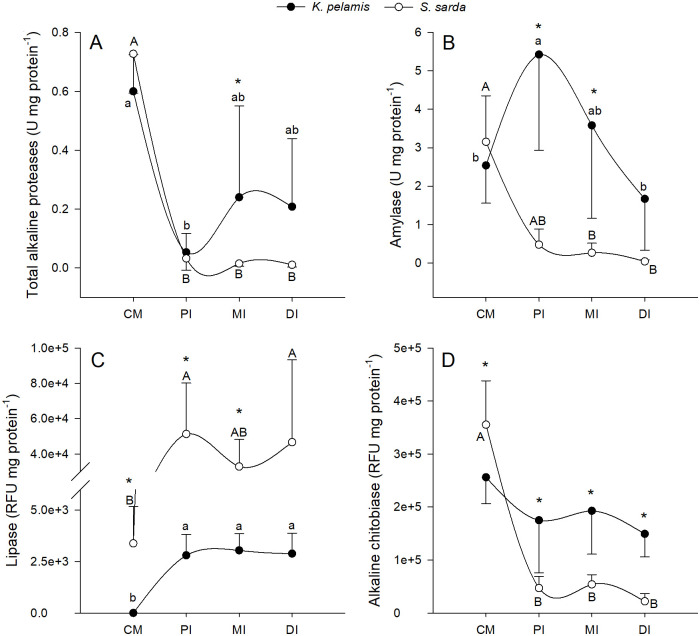
Alkaline digestive enzymes activity levels. Total alkaline proteases (A), amylase (B), lipase (C) and alkaline chitobiase (D) activities in *Katsuwonus pelamis* (black dots) and *Sarda sarda* (white dots). Data are presented as mean ± SD (*n* = 10, *n* = 5 for distal intestine of *K*. *pelamis*). Different lowercase letters (a,b) represent statistical differences between digestive organs of *S*. *sarda*, while different capital letters (A, B) represent statistical differences between digestive organs of *K*. *pelamis* (P<0.05). Asterisks represent statistical differences between species (P< 0.05). Absence of asterisk indicates no statistical difference between species (P>0.05).

Regarding intestinal digestive enzymes activity levels, results are shown in Fig 5. Overall, *K*. *pelamis* tended to show higher enzymatic activity levels in the intestine, while for *S*. *sarda* the activity was higher in the caecal mass. The only exception to this pattern was for lipases activity, which was higher in *S*. *sarda* in all the tissues analyzed.

The highest total alkaline proteases activity levels were reached in the caecal mass in both species, being statistically higher than in all the intestine segments in *S*. *sarda* (P = 0.001), and only statistically higher than activity in proximal intestine in *K*. *pelamis* (P = 0.003). Different activity levels between species were only detected in the middle intestine (P = 0.048) (Fig 5A).

Inverse amylase activity patterns were observed for both species (Fig 5B). While in *K*. *pelamis* amylase activity peaked in the anterior part of the intestine followed by a gradual decrease in activity along the intestine reaching low activity levels in the distal intestine similar to those at the caecal mass (P = 0.008), *S*. *sarda* showed the highest amylase activity level in the caecal mass followed by a gradual decline of activity along the intestine, recording the lowest activity levels in the middle and distal intestine (P = 0.000). Comparing between species, amylase activity showed different activity levels in the proximal and middle intestine, being statistically higher in *K*. *pelamis* (P = 0.000 and 0.002, respectively).

Lipase activity was statistically higher in all the digestive tissues in *S*. *sarda* than in *K*. *pelamis* (P = 0.014, 0.001 and 0.000 for caecal mass, proximal intestine and middle intestine, respectively), with the only exception of the distal intestine (P = 0.076). *K*. *pelamis* showed significantly higher lipase activity levels in the proximal and distal intestine than in the caecal mass (P = 0.032), while in *S*. *sarda* the highest lipase activity levels were achieved in the intestine (P = 0.003) (Fig 5C).

The activity of alkaline chitobiase was similar between all the digestive tissues in *K*. *pelamis* (P = 0.061), while in *S*. *sarda* it was statistically higher in the caecal mass than in the intestine (P = 0.000). Comparing the alkaline chitobiase activity levels between species, *S*. *sarda* showed significantly higher enzymatic activity levels in the caecal mass (P = 0.013), while by contrast, *K*. *pelamis* revealed higher activity in all the intestine segments (P = 0.005, 0.000 and 0.001 for proximal, middle and distal intestine, respectively) (Fig 5D).

### *Katsuwonus pelamis* gut microbiota composition

Bacterial 16S rRNA V2-V3 regions of 5 samples of the distal intestine of *Katsuwonus pelamis* (SKJ) were analyzed. HTS analysis yielded a total of 868728 raw sequences, after removing chimeric sequences a total of 362721 reads were used for downstream analysis. Data were deposited in the Zenodo Repository: https://doi.org/10.5281/zenodo.4540577 [25].

Alpha diversity parameter results are shown in Table 4. The number of observed OTUs as well as results for Chao1 and PD indexes tended to be higher in the individual SKJ3. By contrast Shannon index was similar between all individuals.

**Table 4:** Gut microbiota alpha diversity indexes.

Sample ID	#Observed OTUs	Chao1	PD	Shannon
SKJ1	147	129.69	5.71	3.49
SKJ2	151	130.00	5.88	5.30
SKJ3	320	235.86	12.99	4.97
SKJ4	187	143.08	7.27	4.02
SKJ5	186	145.24	6.99	3.39

Alpha diversity indexes; Chao1 index, phylogenetically diverse (PD) whole tree and Shannon index (operational taxonomic units [OTUs]) of five individuals of *Katsuwonus pelamis* (SKJ). OTUs are defined at 97% sequence similarity.

**Fig 6:**
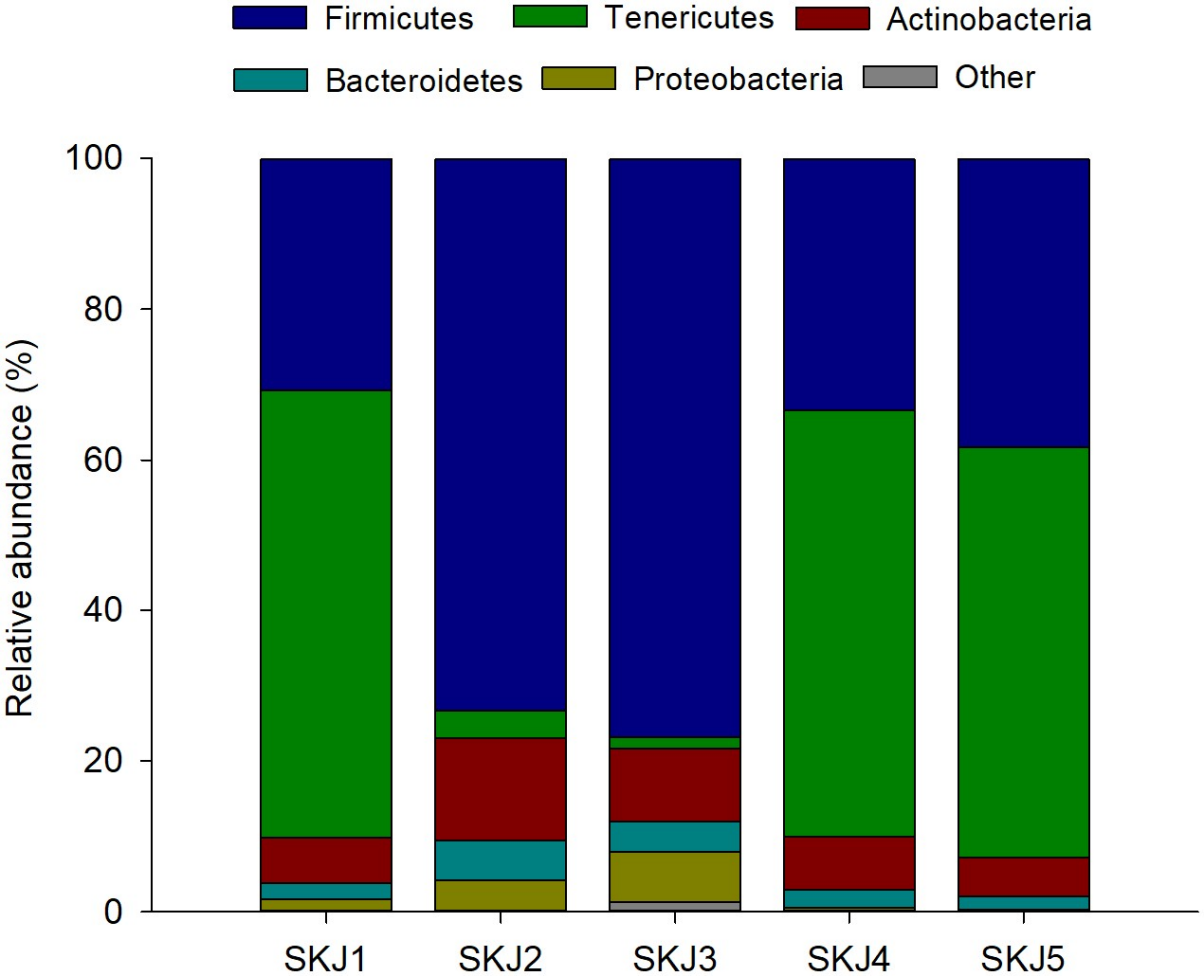
Gut bacterial ecosystem at phylum level. Taxonomic composition of the phyla level (%) of gut microbiota of five individuals of *Katsuwonus pelamis* (SKJ). The plots represent the most abundant representative phylum (> 2%).

The composition of gut bacterial ecosystem is shown at phylum level subdivided by individual samples (Fig 6). The gut microbial ecosystem of *Katsuwonus pelamis* was dominated at the phylum level by *Firmicutes* (average abundance 50.5%), followed by subdominant phyla such as *Tenericutes* (35.2%), *Actinobacteria* (8.3%), *Bacteroidetes* (3.1%) and *Proteobacteria* (2.6%).

The relative abundance of reads assigned to the general level (Table 5) was greatest for *Mycoplasma* (average abundance 35.2%) followed by *Blautia* (9.7%), *Dorea* (6.2%) and *Bifidobacterium* (4.9%).

**Table 5:** Gut bacterial ecosystem at genus level.

Sample ID	Mycoplasma	Blautia	Dorea	Bifidobacterium
SKJ1	59.41	2.56	6.18	3.00
SKJ2	3.74	6.44	14.40	6.84
SKJ3	1.57	8.88	2.94	5.82
SKJ4	56.63	12.33	3.14	4.88
SKJ5	54.44	18.45	4.29	3.73

Gut microbiota at genus level of five individuals of *Katsuwonus pelamis* (SKJ). Values are relative abundance (%). Table shows the four most abundant representatives genera.

## Discussion

The present study aims to describe and compare the digestive physiology of skipjack tuna and the Atlantic bonito in a specific habitat, the Gulf of Cádiz. Ten fish of each species were obtained in August 2019, fish were weighed and measured, and body and GIT tissues were sampled for proximate composition and digestive physiology, respectively.

Fish proximate composition is affected by several factors. Environmental changes such as water temperature, diet composition, and fish activity patterns (e.g. migration) are known to influence the composition of fish muscle [26,27]. The values obtained in the present study for proximate composition were within the reported ranges for fish, and specifically, with those previously reported for both species [28–32]. The only exception was for ash content, which was higher in the present work (on average, 4.86 and 1.68%, current and previous data, respectively). This increment might be explained by the fact that ash content is related to the proportion of bones in the sample [33]. For this study, the entire dissected fraction was homogenized for proximate composition analysis, without excluding the vertebral spine, which increased fishbone content in the sample if compared with studies using only the muscle filet. Comparing between the targeted species in this study, moisture, ash and protein content was similar between skipjack tuna and the Atlantic bonito. The fat and protein content of a fish are the main parameters to consider when evaluating the animal nutritional condition [34]. With an average of 23% of protein, results support a higher protein content of tunids when compared with other marine fish species, such us the Bombay duck (*Harpadon nehereus*) or the unicorn cod (*Bregmaceros mcclellandi*), with 10 ± 2.0% and 15 ± 1.1% of protein, respectively [35], standing out as an excellent source of high-quality protein. Regarding lipids content, the skipjack tuna showed significantly lower amount of lipids, with more than 2-fold decrease when compared to the Atlantic bonito. The lower content of lipids in the skipjack tuna might be explained by the highly migratory nature of this species. It has been reported skipjack tuna migrations to the western Mediterranean Sea in summer for spawning [36]. Thus, the loss of energy density may be a result of depletion of lipid reserves to satisfy the migratory energetic demands. Fishes containing more than 2% fat content are commonly grouped under fatty fish [37]. Therefore, according to the results recorded in this study, the Atlantic bonito in the Gulf of Cádiz can be considered a fatty fish, as tunids in general [32], while skipjack tuna cannot. This classification of the Atlantic bonito regarding the lipid content might be attributed to its diet, which consists mainly in schooling lipid-rich fishes, such as anchovy and horse mackerel, although the prey species are site-dependent [14,26,31,38,39]. Until the date, there are no reports describing the feeding habits of the Atlantic bonito in the Gulf of Cádiz.

Both, feeding habits and gut anatomy have consequences in the mode of digestion. Physiological values of pH in digestive organs allows an adequate digestion of dietary nutrients by providing the optimal environment for the activation and action of digestive enzymes [40]. For vertebrates, two gastric acidification strategies have been reported. One is to maintain a permanent acidic environment in the stomach that increases following feeding due to mixing of ingested feed and water with the acidic stomach fluid. This strategy allows rapid digestion processes to occur following a feeding event. The other is to maintain a neutral pH in the lumen of the stomach between meals followed by a decline after feed ingestion, associated to infrequent feeding events [2,41]. Most teleost display this second strategy [38,42–44], while the first one being only reported, up to date, for rainbow trout (*Oncorhynchus mykis*s) [45], cobia *(Rachycentron canadum*) juveniles [2] and southern bluefin tuna (*Thunnus maccoyii*) [41]. With the exception of this last study, there is scarce information for tuna GIT pH dynamics. Results from the present study suggest that both species, the skipjack tuna and the Atlantic bonito, follow the second strategy described above, with a neutral gastric pH between meals followed by a decline after feeding. This hypothesis is supported by the higher pepsin activity recorded at acidic conditions, confirming the need of low pH for pepsinogen activation. The opposite pattern was described by Leef et al. [41] for southern bluefin tuna, with gastric pH ranging from 3.98 to 2.31 with presence and absence of feed in the stomach, respectively. These results revealed the different acidification strategies adopted by species from the same family, probably as results to the adaptive response to different feeding habits. Regarding the alkaline digestion organs, caecum and intestine, pH values in the former were similar between species, being also in line with the values reported for southern bluefin tuna [41]. pH values in the intestine of skipjack tuna trend to be higher in all the segments compared to the Atlantic bonito. Together with the organ-somatic index and enzymatic results, it seems that the intestine is very important for digestion in skipjack tuna, maybe more so than it is in the Atlantic bonito.

The high versatility in fish feeding habits is reflected in different anatomical and functional features. This plasticity allows fish to explore a wide range of feed resources and maximize of its availability in the environment [5]. This is more noticeable in migratory species, which are submitted to variations in their dietary composition while passing through geographical regions. The analysis of digestive enzyme activities provides information on fish nutritional physiology and on fish ability to take advantage of the different nutrients of the diet. As previously discussed, enzymatic activity is highly dependent on pH, thus, the pH of buffers used in the activity measurement will directly influence enzymatic results. In the present study the pH of the buffers was adjusted to the pH recorded for each organ, ensuring that the measured activity reflected the enzymatic levels at sampling time. Pepsin is the major digestive enzyme in stomach, responsible for protein hydrolysis. It is secreted as its precursor, pepsinogen, which converts to its active form, pepsin, in acidic environment. Results from the present study confirm the need of acidic gastric pH for pepsinogen activation in both species. Total pepsin activity levels consider both, pepsin already active at sampling time (equivalent to the activity measured at real pH), but also the activity of force-activated pepsinogen. Pepsin activity pattern in skipjack tuna is in line with the pepsin characterization performed by Nalinanon et al. [46] for this species. Skipjack tuna pepsin was highly active between pH 1.5 and 3.0, while pH range of 6–8 caused enzymatic denaturalization and, as consequence, a notable loss of activity. Proteolytic capacity, both acidic and alkaline, was similar between skipjack tuna and the Atlantic bonito. It is widely accepted that carnivorous fish have higher proteolytic activity than other fish with different feeding habits [47]. In this study, average proteolytic activity was higher in the caecum mass, suggesting that caecum was the most predominant location of protein digestion in the GIT of both species. Results suggest that pepsin is probably responsible for the earlier phase of protein hydrolyzation, being further completed in the caecum by alkaline proteases. Proteolytic activity declined in the intestine, being this decrease more noticeable in the Atlantic bonito intestine. This alkaline proteolytic pattern agrees with the activities of alkaline proteases in other tuna species, which appear limited to either the caecum, as in the bluefin tuna [48], or the caecum and the intestine as in the Pacific bluefin tuna [49].

Chitin, a polymer of N-acetylglucosamine (NAG), is the major structural component of the exoskeleton of arthropods. Digestion of chitin to NAG is carried out by two chitinolytic enzymes; firstly, chitinase cleaves chitin into oligomers of NAG, which are further hydrolyzed by chitobiase, also named NAGase, to its soluble absorbable monomer, NAG [50]. It is considered that the chitinous envelope of prey disrupted by gastric chitinase is further decomposed by chitobiase in the stomach and/or intestine [51]. The biochemistry of fish stomach chitinase was reviewed by Ikeda et al. [52], which described the optimum pH in the acidic region (pH 2.0–5.0). In the present study, both species showed chitobiase activity in the stomach, being significantly higher in neutral than in acid pH environment. Together with what showed for acidic protein digestion, results suggest that nutrients digestion in the stomach of the skipjack tuna and the Atlantic bonito might happen in two phases; the first one activated by pH decline after feed ingestion, in which protein and chitin hydrolysis occur, followed by a second phase, initiated with the recovery to neutral pH levels in the stomach, triggering chitobiase activity. Except for the caecal mass, skipjack tuna showed higher activity of chitobiase than the Atlantic bonito in all digestive organs. Although fish stomach content is spatial variability-dependent, the predisposition of the skipjack tuna for crustacean consumption has been described in several locations, such as southern and northern California waters [53,54], eastern Indian Ocean [55], Laccadive Sea [56], tropical and subtropical Atlantic Ocean [57], West Africa coast [58] and Balearic Sea [12]. Chitobiase activity tends to be associated with those species consuming chitinous prey and lacking mechanical structures to disrupt their exoskeleton [59,60]. Therefore, the high activity level of chitobiase in this species might be explained as physiological and biochemical adaptations that may enable skipjack tuna to break down the prey exoskeleton. Contrary to what described by Prasertsan and Prachumratana [61], which did not find amylolytic activity in any of the tuna species analyzed, in the present study both species showed amylase activity, with similar activity levels in the caecal mass but significantly higher levels in the intestine of the skipjack tuna. This tendency for crustacean consumption by the skipjack tuna might justify an adaptive high activity of both, chitobiase and amylase, as links in the sequential chain of carbohydrates digestion, from the most complex to the simplest.

Lipids are the most important source of metabolic energy in fish. Depending on their feeding habits, carnivorous fishes, especially those from marine environments, have evolve to capture animals with various lipid level contents, being reflected by the high oil levels that can be achieved by fish such as capelin or herring [62]. Previous reports showed that the primary site of lipid hydrolysis for most fish species appears to be in the pyloric ceca and anterior intestine [63]. In the present study, both species showed higher lipolytic activity in the intestine compared to the caecal mass and, surprisingly, activity was maintained along the intestine. However, information is scarce and more fundamental research into tunids digestive lipases is required, especially for the commercially important species. Since the level of fat in the muscle will be partly dependent upon dietary fat levels [64], together with a higher lipase activity, the present study suggests a high predominance of lipids in the Atlantic bonito diet. This is in accordance with the fact that, in the Strait of Gibraltar and the nearby Alboran basin, the Atlantic bonito has been shown to consume, only or almost exclusively, oily teleost fish such as the Atlantic horse mackerel (*Trachurus trachurus*) [14].

The gut microbial community can respond to a variety of factors affecting the host, including changing environmental conditions such as temperature and salinity, habitat, developmental stage, digestive physiology, and feeding strategy [65]. Specifically, trophic level, habitat and possibly host phylogeny are the most likely factors influencing fish gut microbiota composition [66]. Knowledge of the principal composition of fish gut microbiota and understanding the role they play in digestion and whole body function is critical. Regarding trophic level, herbivore fish species revealed a higher characteristic dominance on gut microbial communities when compared to non-herbivorous trophic levels. While the phylum Firmicutes dominate the gastrointestinal microbial communities of herbivores, Proteobacteria is often the dominant phylum at the non-herbivorous trophic levels [67]. Specifically, for scombrids, scarce information is available about gut microbiota composition. Alpha-diversity indexes values in the present study are in line with those described previously for other scombrids, such Spanish and king mackerel (*Scomberomorus maculatus* and *Scomberomorus cavalla*, respectively) [65] and southern bluefin tuna [68]. Ley et al. [69] described an inverse relation between gut microflora phylogenetic diversity and trophic level, being diversity higher in herbivorous than in carnivores’ organisms. However, alpha-diversity indexes in skipjack tuna are higher than values described for top piscivores species (e.g. sharks) [65], suggesting that the skipjack tuna may present a more varied diet, including fish but also crustaceans. Although species-specific selection is proposed as the main factor driving the intestinal bacterial community in fish there are other important drivers in wild populations, especially in migratory animals that experience strong environmental perturbations during migration. The relative importance of host-specific selection or environmental factors in determining the composition of the intestinal microbiome in wild vertebrates remains poorly understood. The present study suggests that within-species variability in the composition of the gut microbiome is also significant (i.e. SKJ2 and SKJ3). Although all samples shared a core microbiota dominated by Firmicutes, Tenericutes and Actinobacteria, the relative abundance of Firmicutes was higher in SKJ2 and SKJ3. This variability might respond to external factors such as temperature, diet, or a combination thereof linked with the historical trajectory of each fish. Results suggest that species-specific selection, but also environmental factors comprise the major drivers of the intestinal community composition of the skipjack tuna. This intra-specific variability in the bacterial relative abundance has been also described for other fish species such as the grey mullet (*Mugil cephalus*) [70] and codfishes [71]. Tenericutes has been also identified as the dominant gut community in the southern bluefin tuna and king mackerel, while Firmicutes in Spanish mackerel [65,68], suggesting that both phyla are part of the microbiota of scombrids. At genus level, gut microbial composition had higher proportion of Mycoplasma (Tenericutes Phylum), followed by Blautia and Dorea (Firmicutes phylum) and Bifidobacterium (Actinobacteria phylum). Mycoplasma are commonly found in fish guts [72]. On the other hand, although Blautia and Dorea are not commonly described as part of the predominant fish gut microbiome genera, the incorporation of oligosaccharides in European sea bass (*Dicentrarchus labrax*) diet increased Blautia [73], and the same effect has been observed for Dorea in mice [74] suggesting a link between these genus and dietary carbohydrates. These results suggest a modulation of the skipjack tuna gut microbiota composition by its feeding habits, which suggests a potential relationship between the abundance of these bacteria and crustacean exoskeleton digestion in this species.

In conclusion, the digestive physiology of skipjack tuna and the Atlantic bonito reported in this study contribute to advance in the knowledge on the feeding ecology and trophic niche of both species in the Gulf of Cádiz, as well as on the feeding behaviour and physiology diversity as a result of adaptation to the specific conditions of a particular habitat and season. The integration of the results suggested a clear trophic niche segregation between both species. Stomach pH values were associated in both species with infrequent feeding events. Body proximate composition and digestive lipases activity patterns pointed a high predominance of lipids in the Atlantic bonito diet, results supported the skipjack tuna as a highly opportunistic feeder with a more varied diet, including fish but also crustaceans as prey. The gut microbial community in this species is dominated by Firmicutes and Tenericutes at phylum level, and by Mycoplasma, Blautia and Dorea at genus level.

## Supporting information

S1 Fig Selected regions of the gastrointestinal tract for digestive enzymes analysis (picture from a *Sarda sarda* sample). (TIF)

S1 Table Presence of feed in the digestive organs of *Katsuwonus pelamis* (SKJ) and *Sarda sarda* (ATB) at sampling. (DOCX)
