# Supplementary figures and images for: Characterization and comparison of the digestive physiology of two scombrids, Katsuwonus pelamis and Sarda sarda, in the Gulf of Cádiz
Source: PLoS One. 2021 Apr 14;16(4):e0249541. doi: 10.1371/journal.pone.0249541 (PMC8046184; doi:10.1371/journal.pone.0249541)

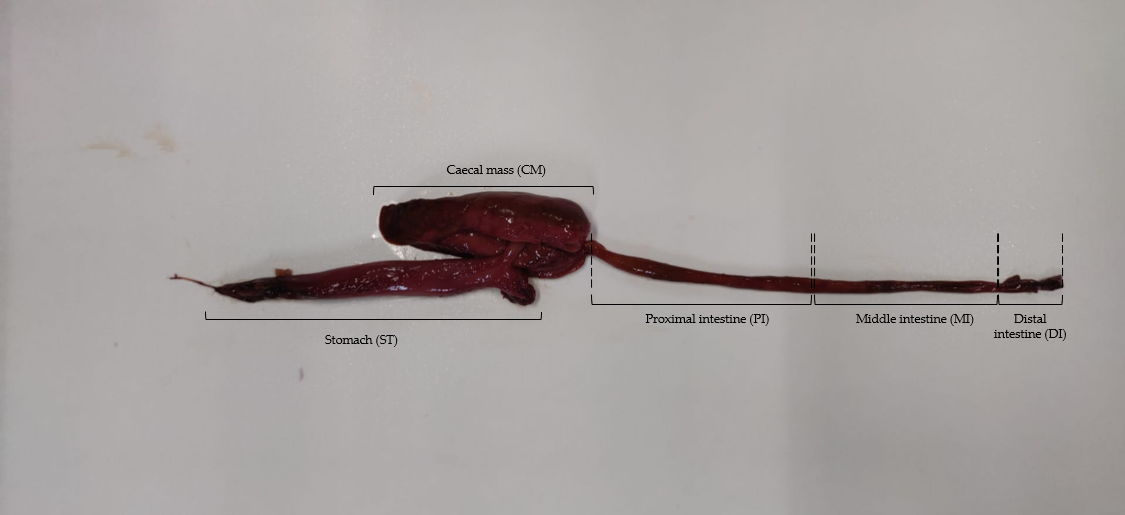

Supplement: S1 Fig — (TIF) [file pone.0249541.s001.tif]
