# Supplementary material for: Characterization and comparison of the digestive physiology of two scombrids, Katsuwonus pelamis and Sarda sarda, in the Gulf of Cádiz
Source: PLoS One. 2021 Apr 14;16(4):e0249541. doi: 10.1371/journal.pone.0249541 (PMC8046184; doi:10.1371/journal.pone.0249541)
Supplement: S1 Table — (DOCX) [file pone.0249541.s002.docx]

Table S1. Presence of food in the digestive organs of sampled individuals of *Katsuwonus pelamis* (SKJ) and *Sarda sarda* (ATB).

| Individual | Stomach | Intestine | | |
| --- | --- | --- | --- | --- |
|  |  | Proximal | Middle | Distal |
| SKJ1 | - | +++ | + | - |
| SKJ2 | - | +++ | +++ | + |
| SKJ3 | - | +++ | ++ | - |
| SKJ4 | - | +++ | +++ | - |
| SKJ5 | - | +++ | +++ | + |
| SKJ6 | - | +++ | +++ | + |
| SKJ7 | - | +++ | +++ | - |
| SKJ8 | - | +++ | ++ | - |
| SKJ9 | - | +++ | + | - |
| SKJ10 | - | +++ | +++ | - |
| ATB1 | - | +++ | +++ | + |
| ATB2 | - | ++ | +++ | - |
| ATB3 | - | + | +++ | + |
| ATB4 | - | +++ | +++ | - |
| ATB5 | + | +++ | +++ | - |
| ATB6 | - | +++ | +++ | ++ |
| ATB7 | - | + | +++ | + |
| ATB8 | + | ++ | +++ | - |
| ATB9 | - | +++ | +++ | - |
| ATB10 | - | +++ | +++ | +++ |

Visual evaluation of presence of feed was performed by the same observer in all samples for reduction of possible bias. Categories were set as follows: empty (-), residual food (+), partially full (++) and full (+++).
